# Forensic psychiatry revisited

**DOI:** 10.4103/0019-5545.69258

**Published:** 2010-01

**Authors:** S. Nambi

**Affiliations:** Department of Psychiatry, Sree Balaji Medical College and Hospital, Chennai - 600 044, India

**Keywords:** Forensic psychiatry, mental health legislation, psycho-criminology

## Abstract

Articles related to Forensic Psychiatry published in the Indian Journal of Psychiatry and Neurology and Indian Journal of Psychiatry during the last 60 years revisited. During these years, around 50 articles have been published in this subject. Psycho-criminology is the theme dealt with in most of the articles. Highlights of some of the important articles are mentioned.

## INTRODUCTION

It is a great opportunity to review the articles related to forensic psychiatry, which were published in the Indian Journal of Psychiatry (IJP) during last 60 years.[1]

In the modern days, almost every aspect of life is regulated or affected in some way by law. All civilizations in the world have enacted laws to regulate human behavior, so that even the weakest can live freely and enjoy all the human rights. Legislations form the important component in the implementation of mental healthcare. There is a dynamic relationship between the concept of mental illness, the treatment of the mentally ill and the law.

“Forensic Psychiatry is a poorly defined specialty with little organized training in most countries.” (WHO working group) Working knowledge of the law that regulates the practice of psychiatry assists clinicians in providing good care to patients. Psychiatrists cannot be expected to be as knowledgeable of the law as lawyers, but they do need to understand how the law and psychiatry interact in various common clinical situations.[2]

In reviewing the forensic-related articles published in the Indian Journal of Psychiatry (IJP) during last 50 years, it is noted that there are only around 50 articles cited. If we were to classify these articles, they would fall under the following three heading:

1. Psycho-criminology related articles - there are around 38 articles.
2. Mental health legislation articles - numbered around 5 and
3. The others are around eight

There is one letter to the Editor and Book Review for the book titled “*Psychiatry and law*” by J.C.Marfatia in 1972. These are other interesting findings in analyzing these articles. There are totally eight articles related to forensic psychiatry during the past 50 years of IJP, which include one invited editorial. They are:

1. Indian Journal of neurology and psychiatry of October 1949, the editorial titled “*Indian Lunacy act*” (author unknown)
2. B.B. Sethi. “*Cult Of violence*” in 1984
3. B.B. Sethi. “*Need for Growth of Forensic Psychiatry*” in 1984
4. S.M. Channabasavanna. “*Dialogue with Judiciary*” in 1985
5. A.K. Aggarwal. “*Mental Health and the Law*” in 1992
6. T.S.S. Rao. “*Psychiatrist and the Science of Criminality*” in 2007
7. T.S.S. Rao. “*Criminal Behavior - A dispassionate look at parental disciplinary practices*” in 2007
8. A.K. Kala. Invited Editorial titled “*of Ethical Compromising Positions and Blatant Lies about Truth Serum*” in 2007

These are two articles of Murthy Rao oration delivered by R.C. Kapoor in 1994 tilted “*Violence in India - A Psychological Perspective*” and L.P.Shah in 1999 titled “*Forensic Psychiatry in India-Current Status and Future Development*”.[3]

It is heartening to note that the two presidential addresses are in Forensic Psychiatry, the first one by O. Somasundaram in 1987 on “*The Indian lunacy act - 1912*” the historic background; and the second one by S.Nambi on “*Marriage, Mental Health and Indian Legislation*” in 2005. It is to be noted here that the Presidential Address by J.K.Trivedi in 2004 on “*Terrorism and mental health*” is also to be taken into account. Incidentally, both these presidents are from Chennai, South India. At this juncture, it is to be noted that majority of the forensic psychiatry articles in the last 50 years, around 20 are from the South and many of them are from Chennai center. The central zone (Lucknow) followed by north and west zone represent the second, third and fourth in the descending order in this category. Even though the East zone members have done some pioneering work in forensic psychiatry, only a few articles have been published. It is to be noted here that there are three forensic psychiatry articles authored by foreign authors and one by military (Navy) psychiatrists. Apart from the two presidential addresses, specifically in forensic psychiatry, Vidysagar’s Presidential Address in 1973 and V.S.P. Basyam’s Presidential Address in 1997 have some important points mentioned about Forensic Psychiatry and Mental Health Legislation.

When we review the Forensic Psychiatry articles in the IJP articles, we cannot miss one name ie; O. Somasundaram of Chennai. Among all the articles on Forensic Psychiatry published by the IJP, the contribution by O. Somasundaram is nearly one-fourth. His articles include following:

1. Guilty but insane-some aspects of Psychiatric Crime - 1960
2. Insanity versus Criminal Responsibility -1964
3. Psychiatrically Ill Under Trail Patients - 1969
4. Men who Kill their Wives - 1970
5. Crimes of Persons with Epilepsy - 1972
6. The Mothers who Kills their Children - 1973
7. Crimes of Persons with Schizophrenia - 1974
8. Crimes of Persons with Affective Disorder - 1977
9. Murder in Tamil Nadu - a Study of Murder Trial - 1980
10. A Study of Delinquent Boys - 1980
11. The Indian lunacy act - 1912 the historic background: The Presidential Address - 1987
12. The Psychiatry of Assailants of Tamil Nadu Chief Ministers - 1992

In his Presidential Address on “*The Indian lunacy Act-1912 the historic background*,”[4] he traced the evaluation of legislation of various countries with a special mention on British Legislations. At that time, he mentioned that the Indian Lunacy act was out modeled and should be replaced and that there is a need to change the Act for a more comprehensive Mental Health Act.

Nambi, in his Presidential Address in 2005, mentioned the changing concepts of marriages in India with special emphasis on the increased rate of divorce. He narrated the relation between marriage and various mental health problems. The important aspect of his article in “*Marriage, Mental Health and Indian Legislation*” is the various legal procedures outlined by different personal laws in this country. He also mentioned as to how the unsoundness of mind affects the capacity to marry and form a ground for divorce, based on these personal laws prevailing in India.[5]

It is worthy to mention here, two important articles: One by B.B. Sethi[6] in his editorial in 1984 and the other by L.P. Shah in his DLN Murti Oration in 1999. To quote B.B.Sethi “Majority of patients who require the expertise of a Psychiatrist are those who may harm themselves or the society, are not able to look after the welfare of their family or property and may turn out to be dangerous if allowed to act out of their thinking. Their management quite often requires taking away their basic civil rights, involving freedom, activities and residence. According to existing laws in force, such restraints can be imposed only through a due process and thus it is imperative that the psychiatrists be familiar with these”. To quote L.P. Shah, “a large number of mental health, legal and law enforcement professionals are ignorant of mental health related laws. This is indeed a very shocking state of affairs and there is an urgent need to correct the situations” I quote L.P. Shah[3] again, “there is hardly any literature in India, clarifying the topics like negligence, informed consent, confidentiality, certification, seclusion, suicide, homicide and the complication of various therapies.” He also recommended that the level of teaching and research in the area of Forensic Psychiatry be upgraded and the in-service programs for the medical and psychiatric personnel, the judiciary and the law enforcement bodies as well as social welfare organization to protect the Human Rights of the Mentally ill be enhanced.

## CONCLUSION

Forensic Psychiatry is a clinical subspecialty of psychiatry. The subject is concerned with an area where psychiatry and law meet. It overlaps interfaces and interacts with psychiatry and law in all aspects. Law is the sanctioning discipline and psychiatry is the therapeutic discipline. Due to various reasons, Forensic Psychiatry is reared as Cinderella in our country, which is much neglected, ignored, misinterpreted and misunderstood. It is very essential that more and more articles based on the modern concept of Forensic Psychiatry and the practical implementation and difficulties of various laws relating to mental health should be published periodically in the IJP. Of course, there are a few psychiatrists in the country with interest in Forensic Psychiatry, who carry out some work in this area, but the main problem of our psychiatrists is that they do not document or publish it. For example, the authors of this article have presented and published more than 50 articles in various souvenirs, regional journals and in the national and international conferences and CMES. But, hardly only one or two of these have been published. We have to understand that the scientific work which is carried out should be documented or published; if not documented it means it is not done.
